# Phylogeographic Diversity of the Lower Central American Cichlid *Andinoacara coeruleopunctatus* (Cichlidae)

**DOI:** 10.1155/2012/780169

**Published:** 2012-09-12

**Authors:** S. Shawn McCafferty, Andrew Martin, Eldredge Bermingham

**Affiliations:** ^1^Biology Department, Wheaton College, 26 East Main Street, Norton, MA 02766, USA; ^2^Department of Ecology and Evolutionary Biology, University of Colorado, Boulder, CO 80309-0334, USA; ^3^Smithsonian Tropical Research Institute, P.O. Box 2072, Balboa, Panama

## Abstract

It is well appreciated that historical and ecological processes are important determinates of freshwater biogeographic assemblages. Phylogeography can potentially lend important insights into the relative contribution of historical processes in biogeography. However, the extent that phylogeography reflects historical patterns of drainage connection may depend in large part on the dispersal capability of the species. Here, we test the hypothesis that due to their relatively greater dispersal capabilities, the neotropical cichlid species *Andinoacara coeruleopunctatus* will display a phylogeographic pattern that differs from previously described biogeographic assemblages in this important region. Based on an analysis of 318 individuals using mtDNA ATPase 6/8 sequence and restriction fragment length polymorphism data, we found eight distinct clades that are closely associated with biogeographic patterns. The branching patterns among the clades and a Bayesian clock analysis suggest a relatively rapid colonization and diversification among drainages in the emergent Isthmus of Panama followed by the coalescing of some drainages due to historical connections. We also present evidence for extensive cross-cordillera sharing of clades in central Panama and the Canal region. Our results suggest that contemporary phylogeographic patterns and diversification in Lower Central American fishes reflect an interaction of historical drainage connections, dispersal, and demographic processes.

## 1. Introduction

 Species distribution patterns are determined in large part by a combination of ecological (e.g., competition, predation, and demography) and historical (e.g., vicariance and dispersal opportunities) processes. Though in the past there was a general tendency to emphasize the role of ecology in structuring communities, historical processes have received increasing attention of late [1–5]. This is particularly true for the freshwater fishes where dynamic patterns of habitat loss (vicariance) and movement across freshwater connections (dispersal) are key determinants of species distributions [4, 6]. In addition, there is increasing evidence that contemporary patterns in species distributions and phylogeography tend to reflect historical rather than contemporary drainage connections in many freshwater species [7–10]. It is widely accepted that phylogeographic patterns have the potential to yield important insights into the mechanisms driving biogeographic structure. In particular, a close correspondence between intraspecific phylogeographic patterns and biogeographic provinces can be readily explained by historical processes. However, when discordance occurs, we must consider other factors to account for this disparity. Ecological differences in dispersal ability and demographics are two potentially important factors that can lead to differences between biogeographic and phylogeographic association [11].

 The importance of the lower Central American region (LCA, defined here as northwestern Colombia north to Lake Nicaragua) in determining the distribution patterns for many species is well known [12–15]. The rising Isthmus of Panama acted as a corridor for many freshwater fishes, enabling the conquest of Mesoamerica by South American species from northwestern Colombia [4, 16–20]. Smith and Bermingham [4] were able to show that this region is divided into a number of distinct biogeographic provinces based on species presence/absence, and proposed a model of historical patterns of vicariance and dispersal (through stream capture and anastomosis) to explain their results.

 To date, detailed phylogeographic analyses and models of the colonization of LCA by freshwater fishes have focused on primary freshwater fishes (species that are relatively intolerant of seawater) in the Characiformes, Siluriformes, and Gymnotiformes [20–24]. In this paper, we provide a detailed phylogeographic description of a presumptive secondary freshwater fish (species with an elevated physiological tolerance for brackish or salt water), the cichlid *Andinoacara coeruleopunctatus*, in order to test expectations regarding the role of dispersal and diversification based on ecological differences. Our interest in *A. coeruleopunctatus* derives from the fact that despite being considered a secondary freshwater fish species by Myers [25], its distribution is relatively limited (Panama and southern Costa Rica) and occurs commonly in all but one (Bocas del Toro) of the biogeographic provinces defined in southern LCA. In addition, it shares a similar though not identical distribution pattern across LCA as many of the primary freshwater fishes previously used to construct models of colonization in this area [20]. Here, we specifically test the hypothesis that the phylogeographic pattern found in *A. coeruleopunctatus *differs from the biogeographic provinces defined by Smith and Bermingham [4] due to potentially greater opportunities for dispersal among drainages. In addition, we compare these patterns to those found in past studies based on primary freshwater species and put forward an explanation for differences found.

## 2. Materials and Methods

**Figure 1:**
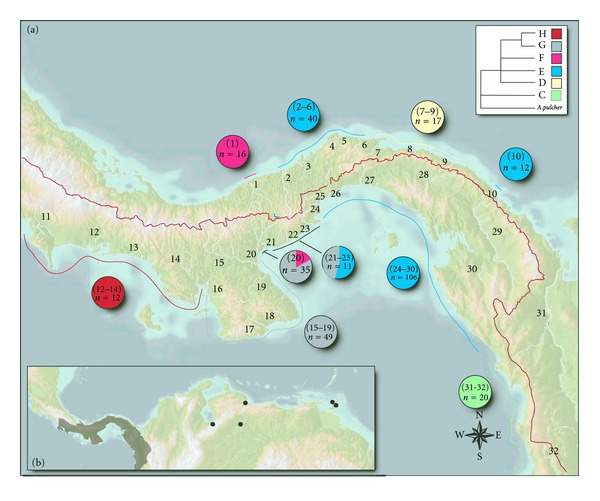
Map showing (a) sampling locations used in this study and (b) the distribution range of *A. coeruleopunctatus*. In addition, the frequency of ATP6/8 clades and the sample size used to estimate clade frequencies are included. Drainage areas and biogeographic regions follow those of Smith and Bermingham [4]: (1) Rio Cocle del Norte; (2) Rio Indio; (3) Rio Chagres; (4) Rio Cascajal; (5) Rio Pina Pina; (6) Rio Cuango; (7) Rio Mandinga; (8) Rio Azucar; (9) Rio Playon Chico; (10) Rio Acla; (11) Rio Coto; (12) Rio Chiriqui; (13) Rio San Felix; (14) Rio San Pablo; (15) Rio Santa Maria; (16) Rio Tebario; (17) Rio Tonosi; (18) Rio Oria; (19) Rio La Villa; (20) Rio Cocle del Sur; (21) Rio Anton; (22) Rio Farallon; (23) Rio Chame; (24) Rio Capoeira; (25) Rio Caimito; (26) Rio Grande; (27) Rio Pacora; (28) Rio Bayano; (29) Rio Tuira; (30) Rio Iglesia; (31) Rio Atrato, Colombia; (32) Rio Baudo, Colombia. The biogeographic areas are Chagres: 1–10; Chiriqui: 11–13; Santa Maria: 14–24; Tuira: 25–31. Values within pie charts are the drainage ID numbers used to estimate the frequency and the sample size.

 Samples of *Andinoacara* were collected by electroshocking or seining in various drainages from Costa Rica, the Republic of Panama, Columbia, Venezuela, Trinidad, and Peru.  Figure 1 summarizes drainage locations sampled in this study. The geological history of this area is detailed in Bermingham and Martin [20], while the biogeographic structure is characterized in Smith and Bermingham's [4]. Drainage boundaries follow those of Smith and Bermingham and are specified in Table S1.1 (Supplementary Data available online at doi:10.1155/2012/780169). 

 Preliminary sample identifications were made in the field. Individuals for DNA analysis were tagged and samples collected by excising gill tissue from the right side of the specimen. Gill tissue was preserved in an ambient temperature DMSO/EDTA buffer [26] or in 95% EtOH. The specimens were subsequently preserved in formalin, transferred to 70% ethanol, and deposited in the Neotropical Freshwater Fish Collection located at the Smithsonian Tropical Research Institute in the Republic of Panama [27]. Table S2 lists the STRI identification numbers, drainage locales, and Genbank accession numbers for all samples used in this study.

 DNA sequence data was collected from the mitochondrial ATP synthase 6/8 (ATP6/8) using routine laboratory procedures [28]. The entire ATP6/8 region was amplified and sequenced using primers L8331/H9236/L8524 [28]. DNA sequence was determined using ABI 377 and 3100 automated sequencers following the manufacturers' recommendations.

 For all phylogenetic analyses, *A. biseriatus* and *A. rivulatus *were used as outgroups [29, 30]. Redundant haplotypes were combined into single OTUs keeping track of geographic origin. A maximum likelihood (ML) approach was used to estimate phylogenetic relationships among mtDNA haplotypes using the program GARLI v2.0 [31]. The optimal evolutionary model(s) (those within 95% CI of the optimal) was inferred using jModelTest [32] and PhyML [33] following the author's recommendation. The resulting best models estimated in jModelTest based on the Bayesian Information Criteria (BIC) were used in ML analyses in GARLI with branch support estimated by bootstrapping (*n* = 1000) with the assistance of SumTrees found in the Python package DendroPy [34].

 Additional estimates of clade support were determined by Bayesian inference using the program MrBayes 3.1 [35]. We ran four independent Bayesian analyses for 1,000,000 generations with 4 Markov chains sampling every 100 generations using the GTR+I+G model. The resulting log-likelihood scores were plotted against generation time to search for stationarity in the results. For all analyses, stability was reached within ca. 1,000 generations. The burn-in was set at 1000 generations and the remaining tree samples used to generate a 50% majority rule consensus tree to calculate the posterior probability of each clade.

 In order to boost the confidence in the phylogeographic patterns observed, 318 individuals of *Andinoacara* distributed throughout the LCA region were studied using PCR restriction fragment length polymorphism genotyping (RFLP) similar to that of Reeves and Bermingham [24]. Five restriction enzymes (AluI, DdeI, HaeIII, HinfII, and RsaI) were used that in combination allowed us to uniquely identify samples to one of the primary clades found in the ML analysis. Individuals from each clade that were sequenced were included in the analysis as reference. This allowed us to estimate the frequency of the primary clades within the various drainage areas in Panama.

 To test if the ATP6/8 region was evolving in a clock-like manner, we performed a likelihood ratio test using PAUP* v4.0d64 [36]. A GTR+I+R model was used with parameters estimated as above. Based on these results, a relaxed clock Bayesian estimation of the dates of diversification of the primary clades was performed using BEAST v1.6.2 [37]. The TRN+I+G model was used in four independent runs with a chain length of 10^7^ generations, a burn-in of 10000 generations, and parameter sampling every 1000 iterations using the uncorrelated lognormal distribution option for rate variation across branches. A Yule tree prior was assumed and all other options were set to default. Lacking any well-defined calibration points (i.e., fossil representation) in the resulting phylogeny, we used a per lineage rate of divergence (divergence per lineage per 10^6^ years) estimated from McCafferty et al. [38] of 0.0065 ± 0.002 based on parametric bootstrapping of *Abudefduf saxatilis*/*trossulus* (Pomacentridae) geminate species pair for the ATP6/8 gene region [39]. We feel, this estimate of divergence rate is appropriate for our purposes given that the estimate is from a homologous mtDNA gene region, the value falls squarely in the middle of estimates from other fish species, and that the Cichlidae and Pomacentridae are relatively closely related. The independent runs were combined using LogCombiner v1.4.8, dates of divergence along with their 95% confidence intervals (HPD) were estimated using Tracer v1.4, and the resulting phylogeny and 95% HPD for the dates of divergences for the major clades visualized using FigTree.

## 3. Results

 Complete ATP6/8 gene sequences were determined for 47 individuals of *A. coeruleopunctatus* collected throughout the species' range, plus an additional 8 individuals representing the closely related *A. pulcher* from 5 different geographic locations in Trinidad and Venezuela. The outgroup comprises *A. biseriatus* (*n* = 2) from the Atrato River, Colombia and *A. rivulatus* (*n* = 8) representing four drainages along the Pacific versant of Peru. The final dataset contained a total of 842 bp of sequence data for 65 individuals, of which 173 bp are parsimony informative. All sample identification codes and GenBank accession numbers can be found in Table S1.2 (Supplementary Data). Finally, we used RFLP analysis to genotype 318 individuals from 33 rivers to better estimate the distribution of mtDNA clades within drainages and biogeographic regions in Panama.

**Figure 2:**
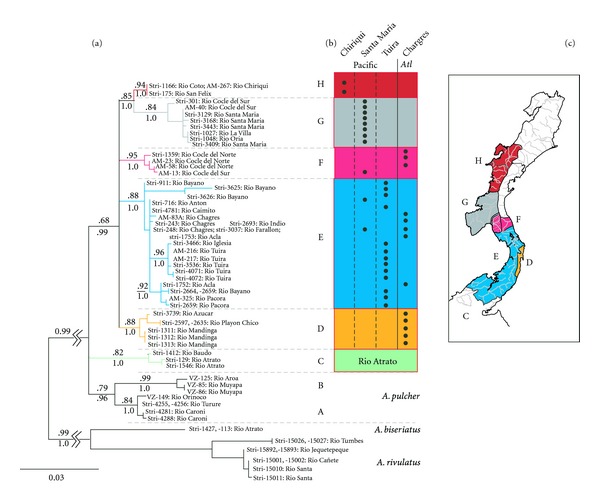
Phylogenetic relationship among mtDNA haplotypes. (a) ATP6/8 ML tree using the TRN+I+G model. Bootstrap support and clade confidence values (posterior probabilities from a Bayesian analysis) for major clades are above and below branches, respectively. (b) Origin of samples by rivers assigned to their biogeographic regions as described in Smith and Bermingham [4]. Branch colors reflect biogeographic regions or drainage areas as found in Figure 1. The inset map is a stylized representation of the range of the major clades along drainage and biogeographic boundaries.

 The resulting ML gene tree for ATP6/8 using the TRN+I+G model (based on the results of model selection using jModelTest; Supplement S1A) is shown in Figure 2. There are several noteworthy features of the topology shown in Figure 2 that can be summarized as follows: (1) the phylogeny identifies eight well-supported mtDNA clades within *A. coeruleopunctatus* and *A. pulcher*; (2) *A. pulcher* forms a monophyletic grouping with two mtDNA clades representing the Orinoco region of Venezuela (labeled clade A) and Rio Maracaibo River/Trinidad (clade B); (3) the relationship between *A. pulcher* and *A. coeruleopunctatus* could not be resolved overall, with a three-way polytomy occurring among *A. pulcher*, a clade consisting of *A. coeruleopunctatus* from the Rio Atrato and Rio Baudo drainages in Colombia (clade C), and the remaining *A. coeruleopunctatus*; (4) *A. coeruleopunctatus* from Panama and Costa Rica form a monophyletic group consisting of five primary clades (labeled clades D through H) with a three way polytomy at its root.

### 3.1. Paraphyly of *A. coeruleopunctatus* with respect to *A. pulcher*

 The polytomy describing the relationship among *A. pulcher*, Colombian *A. coeruleopunctatus*, and the remaining Panama and Costa Rica *A. coeruleopunctatus* appears to be robust based on the results of various phylogenetic analyses (Supplement S1B), and is unchanged by an additional 1048 bp of ND2 sequence (Supplement S1C). In addition, there is no evidence for saturation effects in either the ATP6/8 or combined data sets (Supplement S1F). Therefore, we consider the relationship unresolved; both *A. pulcher* and *A. coeruleopunctatus* from Colombia form reciprocal monophyletic clades that are approximately equally divergent from the remaining *A. coeruleopunctatus* from Panama and Costa Rica.

### 3.2. Phylogeographic Patterns and Cross-Cordillera Exchange

 Following Figure 2, the next principal clade consists of four major clades (D, E, F, and [G,H]) that form an unresolved polytomy among the Panama *A. coeruleopunctatus* though each clade is well supported. Clade D is composed of samples derived from Rio Mandinga, Rio Azucar, and Rio Playon Chico in the Western San Blas region. In most of our analyses, this group forms a poorly supported basal clade leading to the remaining Panama *A. coeruleopunctatus*, with an average bootstrap support of ca. 55% but with 100% support based on the Bayesian analysis. Due to the rather low support from most analyses for a basal D clade, we choose to show clade D as part of a four-way polytomy. Whether one considers this Western San Blas clade, a sister clade to all other Panama *A. coeruleopunctatus* or a member of an unresolved polytomy is not particularly important to the discussion below. The key point is that this region appears to form a unique, reciprocal monophyletic group closely related to all other Panama *A. coeruleopunctatus*.

 The remaining three clades (E, F, and [G,H]) form an unresolved polytomy among the remaining Panama *A. coeruleopunctatus* in all analyses though each clade is well supported. Clade E is composed of samples derived from rivers and drainages in the Chagres, Tuira, and Santa Maria biogeographic regions of Smith and Bermingham [4] and includes samples from both sides of the continental divide including a disjunct population from Rio Acla in Eastern San Blas. Clade F represents haplotypes collected from the Rio Cocle del Norte (1 in Figure 1; Chagres biogeographic region) and Rio Cocle del Sur (20 in Figure 1; Santa Maria biogeographic region). This particular clade shows clear evidence of cross-cordillera sharing of mtDNA haplotypes. Finally, the last two clades (G and H) form a sister group, with samples derived from river drainages found in the Santa Maria (G) and Chiriqui (H) biogeographic regions forming separate well-supported clades. Mapping of these clade distributions can be found in Figure 2.

### 3.3. RFLP Analysis of Clade Frequencies

 We sampled a total of 318 individuals from 33 different drainages for 5 restriction enzymes that in combination permit us to uniquely identify individual samples to one of the primary *A. coeruleopunctatus* clades. The resulting clade frequencies are summarized in Table 1 and graphically in Figure 1. Detailed haplotype frequencies can be found in Supplemental Table S1.3). If a particular biogeographic region contained a single clade (e.g., Tuira and Santa Maria in Table 1), then the data were pooled within biogeographic region. Otherwise, the clade frequencies were pooled by drainage area.

 The RFLP data reinforce and expand on the phylogeographic patterns found for the ATP6/8 sequence data. One hundred and fifty-eight individuals representing 13 rivers in the Tuira and Chagres biogeographic regions, including the Rio Acla in eastern San Blas, all are haplotypes specific to the E clade. Furthermore, the 17 individuals sampled from three rivers in the western San Blas area within the Chagres all contained haplotypes specific to the D clade, and the 16 individuals collected from the Cocle del Norte river on the western edge of the Chagres biogeographic region all carried haplotypes specific to the F clade. The RFLP data also confirm that the Atrato in Colombia, the Santa Maria (49 individuals from 6 drainages), and Chiriqui (12 individuals from three drainages) biogeographic regions in Panama are fixed for mtDNA clades C, G, and H, respectively, with no evidence of interchange between the Santa Maria and Chiriqui regions.

 RFLP analysis of 35 individuals also confirmed that Rio Cocle del Sur, a Pacific slope drainage, carried both clade F (17%), characteristic of Rio Cocle del Norte, and clade G (83%), characteristic of the Santa Maria. Again, these results are consistent with the phylogeny based on mtDNA sequence data and provide strong evidence for the movement of fish between the Cocle del Norte to the Cocle del Sur.

*A. coeruleopunctatus* populations collected from the Rio Anton, Rio Farallon, and Rio Chama drainages (21, 22, and 23 in Figure 1) at the western edge of the Tuira/Chagres biogeographic region contain haplotypes from both the E clade, characteristic of the Tuira and Chagres regions, and the G clade, characteristic of the Santa Maria region, in roughly equal frequencies when pooled (45% and 55%, resp.). This is a finding that would have been missed in the absence of the more complete geographic sample collected for the RFLP analysis.

### 3.4. Relaxed Clock Estimates of the Time of Divergence

**Figure 3:**
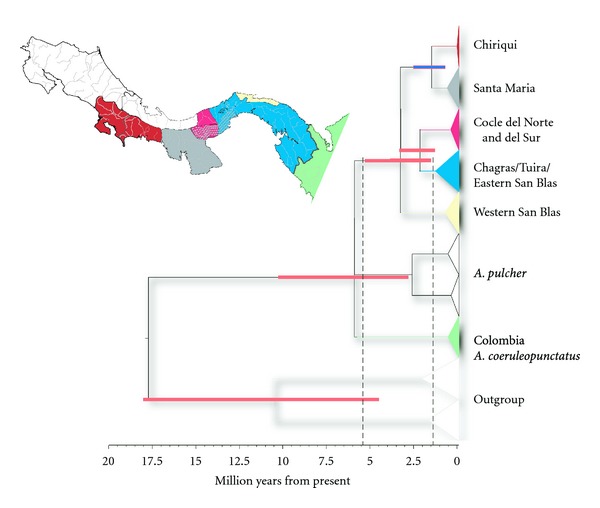
Estimated range of divergence of major clades based on a relaxed Bayesian analysis. Clades are colored as in Figure 1. The inset map is a stylized representation of the range of the major clades along drainage and biogeographic boundaries.

 The estimated dates of divergence based on relaxed clock Bayesian analysis for the major clades are summarized in Figure 3. The MCMC process appeared to perform well with stationarity achieved in all runs and ESS values well exceeding 200. Due to the unresolved relationship among the major clades in Figure 2, our analyses focused on the general time of divergence among these clades rather than the detailed timing of divergence within clades (see Supplement S1E for more information on these results). The estimated date of diversification of the Panama *A. coeruleopunctatus* mtDNA clades is around 3.4 Ma, with a 95% HPD from 1.5 to 5.5 Ma. We estimated the date of divergence among Panamanian A. *coeruleopunctatus*, Colombian *A. coeruleopunctatus*, and *A. pulcher* to have occurred around 5.9 Ma with an estimated HPD of 2.7 to 10.5 Ma.

## 4. Discussion

 The phylogeographic pattern of *A. coeruleopunctatus* in the LCA region was described above based on mtDNA sequence and RFLP data. There are four aspects of our results that are most relevant to our discussion of the relationship to biogeographic patterns and previous work on primary freshwater fishes in this region. First, including *A. pulcher*, the eight unique clades (two in *A. pulcher*, one from *A. coeruleopunctatus* in Colombia, 5 in Panama *A. coeruleopunctatus*) detected are closely associated with the biogeographic regions described by Smith and Bermingham [4] and Abell et al. [40]; however, there are some important differences. Second, the branching order of four of the principle Panama clades was unresolved, suggesting a period of rapid diversification of colonizing populations followed by allopatric differentiation. Third, the timing of this rapid diversification based on a relaxed clock analysis corresponds to the final rise of the Isthmus of Panama in the late Pliocene. Fourth, there is strong evidence for historical cross-cordillera exchange in at least two regions of LCA: one centered in the Cocle del Sur/Cocle del Norte drainages, the other in the Chagres/Tuira region. Before we proceed to discuss the implications of these findings, it is worth considering the placement of *A. pulcher* with respect to *A. coeruleopunctatus*.

### 4.1. Paraphyly of *A. coeruleopunctatus*

 Our results indicate that the relationship among *A. pulcher*, *A. coeruleopunctatus* from Colombia, and Panama *A. coeruleopunctatus* is best characterized as a three-way polytomy, implying that either *A. pulcher* may require reconsideration as a separate species, or *A. coeruleopunctatus* from the Atrato and Baudo River drainages be elevated to specific status, changing the distribution of *A. coeruleopunctatus* to strictly Panama and southern Costa Rica to make it a monophyletic group. Though we strongly emphasize that these results are based on a single mitochondrial gene and therefore should be viewed with caution, they are generally consistent with recent detailed studies on neotropical cichlids that included members of the genus *Andinoacara* [29, 30], though these studies did not include *A. coeruleopunctatus* from Colombia. The unresolved branching order among these three clades dates to between 2.7 and 10.5 Ma with an estimated mean of 5.9 Ma, suggesting that *A. coeruleopunctatus* originated either in northern Colombia or in areas within an evolving LCA landscape itself prior to the estimated final rise of the Isthmus of Panama. Unfortunately the results of our Bayesian clock analysis do not exclude the possibility of a pre-Pliocene dispersal event, though our estimate suggests a much later origin than estimated for secondary freshwater fishes in these regions (ca. 18 to 15 Ma; [20, 23, 41–43]).

### 4.2. Phylogeographic Patterns within* A. coeruleopunctatus* and Biogeography

 One of the key results from our study of *A. coeruleopunctatus* in LCA is that we find significant phylogeographic structure. To summarize, based on both DNA sequence and RFLP data we detected the presence of six unique clades that are consistent in many respects with the biogeographic provinces described by Smith and Bermingham [4]: clade C is from the Atrato region of Colombia, E combines the Chagres, Tuira, and portions of the Santa Maria regions, G is Santa Maria-specific, and H is from the Chiriqui region including Costa Rica. However, there are some key differences that may lend important insights into the forces driving phylogeographic and biogeographic structure in this region. First, our results divide Smith and Bermingham's Chargres region into three groups: (1) a reciprocal monophyletic Western San Blas clade (D), (2) a clade (F) that contains samples from Rio Cocle del Norte (Chargres) and Rio Cocle del Sur (Santa Maria), and (3) the remaining drainages from the Chargres region that all fall within a single clade (E) that includes samples from the Tuira region and two rivers from the Santa Maria (Rio Anton and Rio Farallon). It is interesting to point out that these two rivers are located on the border region between the Tuira and Santa Maria assemblages in Smith and Bermingham [4] and along with Rio Chama appear to represent a transition zone between the Tuira/Chagres and the Santa Maria biogeographic zones on the Pacific coast. The western extent of the Tuira/Chargres clade appears to be in Rio Anton, as there is no evidence for clade E haplotypes in Cocle del Sur. Smith and Bermingham [4] discuss the difficulties of assigning biogeographic boundaries in this area, further highlighting the similarity between the molecular and biogeographic patterns. In summary, our results coalesce the Tuira and Chargres biogeographic zones, excluding the rivers from the Atlantic versant found in Western San Blas and Cocle del Norte, and defines a fuzzy transition zone between the Tuira and Santa Maria assemblages centered around Rio Anton.

 This clear association between phylogeography and biogeography is punctuated by the general unresolved branching order among the clades. A general lack of resolution of branching order among otherwise well supported clades is observed in most of the LCA freshwater fishes studied to date [20, 23, 24] and is not due to saturation effects (Supplement S1F). Whether this basal polytomy is due to rapid diversification or simply reflects the limits of resolution of our data remains to be tested. Nonetheless, the rate of diversification of lineages in the newly emergent landscape must have been quite fast. Estimates of the timing of this diversification event based on a relaxed molecular clock are slightly older but nonetheless still consistent with the estimated date for the final rise of the Isthmus of Panama at 3.2 Ma [14] and are consistent with previous results reported for other freshwater fishes in this region. Though recent evidence suggests a more fully emergent LCA earlier then previously estimated [44], the final closure of the seaway connecting the Pacific and Caribbean remains as estimated in Coates and Obando [14], and appears to have been an effective barrier to the migration of many freshwater species. However, once a newly emergent bridge connected South America with the evolving LCA landscape, it was rapidly colonized by lineages of *A. coeruleopunctatus* that diversified rather quickly in the separate drainage areas.

 Being characterized as a secondary freshwater fish species, we anticipated finding little if any phylogeographic structure among LCA drainages for *A. coeruleopunctatus*. Yet we find strong evidence for the formation of clades along biogeographic regions and drainage areas, a pattern characteristic of primary freshwater fishes in this region. This finding suggests that, regardless whether *A. coeruleopunctatus* is considered a primary or secondary freshwater species, similar forces may be acting to drive community composition and phylogeographic structure in freshwater fishes in this region. It also suggests that ecological differences among species may play a lesser role in determining phylogeographic patterns, a topic we will explore further below after discussing other important aspects of the phylogeography in *A. coeruleopunctatus*.

### 4.3. Cross-Cordillera Exchange

 Another notable result from our analysis is a clear pattern of sharing of mtDNA clades across the central cordillera of LCA. We see this in two regions: the Chagres-Tuira area and further west around Cocle del Norte-Cocle del Sur. In both cases the degree of clade sharing is extensive and readily interpreted in light of the local physiography. In Western Panama, the central Cordillera rises steeply to over 2000 meters in most places as a consequence of late Pliocene and early Pleistocene subduction of a low-density Cocos Ridge [14]. The Cordillera gradually diminishes in relief to the east reaching a low of 200 meters near the headwaters of the Cocle del Sur in Central Panama and 100 meters in the region of the Panama Canal. The San Blas region (the Atlantic slope rivers of eastern Panama) and rivers of the eastern Pacific slope of Panama are separated by mountains rising only about 200 to 500 meters. The Chagres-Tuira region then encompasses one of lowest points of the central cordillera around the Panama Canal region, while the Cocle del Sur-Cocle del Norte are in the region of El Valle, an area of ancient volcanic activity. In both cases it is easy to infer processes of drainage rearrangements (stream capture) to account for cross-cordillera exchange.

 Previous studies of other freshwater fishes in this region also show evidence for cross-cordillera exchange in these two regions. In a total of ten species studied to date, we find three with unequivocal evidence for cross-cordillera sharing of highly divergent haplotypes: *Bryconamericus emperador*, *Brycon argenteus, *and* Rhamdia guatemalensis* [23, 24]. In addition, three species provide limited evidence (due to small sample sizes or phylogenetic branching patterns) for cross-cordillera sharing of clades: *Hypopomus*, *Roeboides*, and *Rhamdia laticauda* [20, 23]. Four of the ten species show no evidence for cross-cordillera exchange: *Pimelodella* (both the A and B types), *Brycon striatulus*, *Bryconamericus scleroparius*, and *Cyphocharax magdalenae* [22, 24].

 Compared with the above studies, our results for *A. coeruleopunctatus* are unique in that, based on large sample sizes from both the mtDNA sequence and RFLP analysis, we are able to make a strong statement concerning the extent of cross-cordillera exchange. We found that not only is cross-cordillera exchange extensive in central Panama and the Canal area, but also there is a key difference in the pattern of clade sharing between the two regions, with important implications. In the Chagres-Tuira area, there is evidence for extensive sharing of a diverse clade (E) by both Atlantic and Pacific drainages (except for western San Blas, which we discuss below), suggesting a long history of dispersal among drainages in this area.

 This is in stark contrast to the situation in the central Panama. In the Cocle del Norte Atlantic drainage, there is evidence for a single clade (F) only, while the Cocle del Sur Pacific drainage contains two clades in very different frequencies, the F clade characteristic of Cocle del Norte (17%) and the G clade (83%) characteristic of the adjacent Pacific biogeographic area of Santa Maria. It is interesting to note that even though the Rio Anton drainage is immediately adjacent to the Cocle del Sur drainage, there is no evidence for sharing the Atlantic derived Cocle del Norte clade (F) in Rio Anton based on both sequence and RFLP data. One possible explanation for this result is that cross-cordillera exchange has occurred by one-way invasion of the Pacific Cocle del Sur by Atlantic Cocle del Norte lineages and that this migration event was relatively recent (occurring well after the final rise of the Isthmus perhaps due to tectonic events in the El Valle area) and limited in extent.

 We attempted to test this hypothesis using a Baysian approach implemented in the program Migrate-n (Supplement S1D). However, the results from a series of analyses were contradictory and unconvincing. Maximum likelihood estimates of migration between Cocle del Norte (CdN) and Cocle del Sur (CdS) were consistent with the above hypothesis (*M*_CdN^−^_ > *M*_CdS_ = 85.030; *M*_CdN^−^_ > *M*_CdS_ = 0), though we have little faith in these estimates. Further Baysian analyses proved suspect, as the data appeared to be insufficient for the analysis to reach stationarity in the estimates of the parameters theta or *M* in most models (Supplement S1D). A test of three competing models of migration between the two regions (model 1 : CdN to CdS only; model 2 : CdS to CdN only; model 3: equal migration) using Bayes Factors suggested that model 1 (CdS to CdN only) had the highest probability, contrary to the ML estimates and our intuitive interpretation.

 Our interpretation of these conflicting results is that our dataset is simply insufficient for this type of analysis and that the results from the Bayes factor approach should be viewed with caution. Nonetheless, determining the migration patterns in this region is important to understand the history or migration in this important region of Panama (see Supplement S1D).

 In stark contrast to this extensive sharing of clades across the cordillera found in central Panama and the Chagres region, we find that the western San Blas region, represented by the Rios Azucar, Mandinga, and Playon Chico, maintains a reciprocal monophyletic clade (D) that roughly forms a polytomy with the other major clades characterizing *A. coeruleopunctatus* in the LCA. There is mixed evidence for this pattern in other neotropical freshwater fishes. *Pimelodella*, *Roeboides*, and *Bryconamericus emperador* evidence a reciprocal monophyletic group in the western San Blas area [20, 22, 24], while *Rhamdia guatemalensis*, *Hypopomus*, and *Brycon argenteus* join western San Blas with the Tuira or Chagres biogeographic region [20, 23, 24]. Smith and Bermingham [4] found no evidence for a separate western San Blas biogeographic province, subsuming it under the Chagres. Nonetheless, the evidence in *A. coeruleopunctatus* for a distinct, allopatric western San Blas clade is unequivocal.

### 4.4. Relationship to Historical Drainage Patterns

 Previous studies on other LCA primary freshwater fishes studied to date suggest that the observed phylogeographic patterns are driven at least in part by historical patterns of river anastomosis (dispersal) and stream capture (vicariance and dispersal) driven by tectonic events and eustatic sea level changes. Though we lack a detailed paleodrainage model for Panama, Smith and Bermingham [4] were able to make good first-order approximations of historical drainage patterns and suggest a model of river anastomosis and stream capture that are consistent with the phylogeographic patterns found here with three important differences. First, cross-cordillera exchange via stream capture may well explain the presence of a distinct Atlantic Cocle del Norte clade that shares haplotypes with Pacific Cocle del Sur. Second, the coalescing of Chargres and Tuira biogeographic regions is also possibly explained by cross-cordillera exchange perhaps enhanced by the construction of the Panama Canal [45]. Finally, the presence of a reciprocal monophyletic WSB clade within the Chargres biogeographic region suggests long-term isolation of this region from surrounding drainages.

 It is difficult to determine why the Tuira/Chargres clade was able to expand into many of the Atlantic drainages yet not into the Cocle del Norte or the Western San Blas region. There are no obvious geological features that might serve to isolate these two regions from the surrounding areas other than a limited continental shelf, yet these two regions retain deep monophyletic lineages dating to the rise of the isthmus. Why would we see the expansion of clades into some regions (e.g., into eastern San Blas and Atlantic drainages from Chagres) but not others that are geographically proximate (e.g., western San Blas) when no clear barrier to dispersal is obvious? The remainder of this paper will explore a possible explanation for the long-term retention of deep monophyletic lineages even when barriers to dispersal appear limited.

### 4.5. Retention of Long-Term Phylogeographic Patterns

 To summarize, we have shown that the phylogeographic pattern found in *A. coeruleopunctatus *can be explained at least in part by historical drainage patterns driven by a combination of eustatic sea level changes and drainage rearrangements consistent with the biogeographic assemblages described in Smith and Bermingham [4] and consistent with the model first proposed by Bermingham and Martin [20]. However, there are aspects of the phylogeographic relationships that are not easily explained by drainage history alone. In particular, though there appear to have been extensive opportunities for dispersal among drainages following the rise of the isthmus, we see the retention of relatively deep monophyletic lineages in some drainage regions that appear to have no distinctive geological barriers to dispersal. This is somewhat of a paradox; how can deep monophyletic lineages be retained despite repeated and periodic opportunities for dispersal? The simplest explanation is of course that dispersal simply did not occur into these areas for some reason. We do not find this a particularly satisfying explanation. We suggest that there may be other mechanisms acting to retain (reciprocal) monophyly even in the presence of dispersal events.

 Bermingham and Martin [20] argue that lineage turnover and ecological differences among species explain in large part the differences in phylogeographic patterns found among primary freshwater fishes in this region. Reeves and Bermingham [24] expanded on this and argued that demographic processes can also have a profound impact on the phylogeographic patterning in colonizing species. The basic thrust of their explanation is that once resident populations (the descendants of early colonizers) approach an environment's carrying capacity, those populations will tend to be more resistant to the influx of new immigrants, a process previously described as persistent founder effects [46] or priority effects.

 Boileau et al. [46] first evoked priority effects to explain observed high levels of divergence among freshwater invertebrate populations in the presence of high rates of dispersal, demonstrating that high Fst values can be retained over long periods of time in populations at or near their carrying capacity even when dispersal rates were substantial; in effect, dispersal becomes effectively decoupled from gene flow. De Meester et al. [47] expanded on this concept, pointing out that adaptive divergence among populations can reinforce priority effects, a process they refer to as the monopolization hypothesis. Though priority effects are commonly put forward as an explanation for the long-term maintenance of phylogeographic variation in freshwater invertebrates (e.g., [47–50]; though see [5]), they are rarely evoked for vertebrates. There is clear evidence for the important role played by priority effects in driving community composition (e.g., [51–55]), yet very limited empirical evidence for its role in maintaining phylogeographic structure [56]. Nonetheless, if a population is at or close to its carrying capacity, then priority effects can potentially play a role in determining long-term patterns of divergence. Waters [57] recently made a similar argument, pointing out the important role that competitive exclusion of secondary dispersers (similar to the monopolization hypothesis mentioned above) may play in maintaining phylogeographic structure within species even in the face of dispersal.

 In the case of *A. coeruleopunctatus* and primary freshwater fishes in this region, we propose that contemporary phylogeographic patterns are determined by a combination of historical and contemporary drainage patterns and demographic effects. When population size is below carrying capacity, then drainages may be susceptible to invasion by migrant lineages that disperse from adjacent drainages during times of river anastomosis and stream capture. If the dispersal event is relatively recent and/or population sizes are relatively high but below carrying capacity, then the presence of multiple divergent lineages can occur, as seen, for example, in the Cocle del Sur drainage. Lineage turnover and local clade extinction can then occur over time, consistent with what we see in the Chagres-Tuira area and eastern San Blas. However, those drainages that retain populations close to the carrying capacity will tend to be resistant to invading lineages during bouts of dispersal. If a particular lineage has an adaptive advantage to invading lineages, then priority effects will be further reinforced (Monopolization hypothesis). This may explain the retention of the reciprocal monophyletic Western San Blas clade in Caribbean Panama and unique clades in the Cocle del Norte and Santa Maria/Chiriqui region.

## 5. Conclusion

 In conclusion, we found significant phylogeographic structure in the LCA cichlid *A. coeruleopunctatus* that is concordant with the biogeographic regions described based on community composition data and consistent with phylogeographic patterns previously described for primary freshwater fishes in this region. However, our results lend a slightly different perspective to previous studies in that we find a coalescing of some biogeographic zones (Chagres/Tuira), evidence for phylogeographic structure within others (Chargres), and strong evidence for cross-cordillera dispersal. Though the branching order of the various phylogeographic clades could not be determined, the date for the diversification of the primary clades is consistent with the final rise of the Isthmus of Panama. We explain our results as due to rapid colonization and diversification in the emerging landscape, with the resulting contemporary phylogeographic pattern a result of both historical drainage connections and demographic processes (i.e., priority effects). This model highlights the dynamic between historical timing, rates of dispersal, and demographic processes with the end result being that priority effects and lineage turnover can potentially have a profound impact on the phylogeographic patterns found in some species. Understanding the extent to which we can begin to tease apart the complex interactions of historical and demographic processes will depend on multispecies phylogeographic studies coupled with careful estimates of rates of dispersal, historical trends in effective population sizes, and statistical testing of these models. Our results with *Andinoacara* demonstrate the added insights into the processes driving phylogeographic patterns that can be gained from studying species with varying life history strategies, ecologies, and phylogenetic histories.

## Supplementary Material

The Supplementary Information file contains tables providing (1) the designation of rivers and drainages into biogeographic provinces (Table S1.1), (2) Genbank accession numbers associated with each sample (pending; Table S1.2), and (3) ATP6/8 haplotype frequencies by river, drainage, and biogeographic region. In addition, the Supplement also provides greater details on (1) choosing the best evolutionary model to use in analyzing the ATP6/8 data set (S1A: Results of jModelTest), (2) additional phylogenetic analyses that were used other than those detailed in the text (S1B: Additional phylogenetic analyses), (3) the resulting phylogeny when an additional 1042bp of ND2 sequence is included with a subset of the ATP6/8 data set (S1C: Additional sequences from ND2 and analyses of the combined datasets), (4) the results for comparison of various migration models using Bayes factors (S1D: Results using Migrate-N), and (5) the results of tests for substitution saturation effects (S1F: Test of saturation ATP6/8).

## Figures and Tables

**Table 1:** Frequency of clades among drainages and biogeographic regions based on RFLP analysis of the ATP6/8 gene region.

River	Biogeographic region or drainage	E	F	G	H	D	C	*n*
Rio Acla	Acla	12						12

Rio Anton	Anton	5		2				7
Rio Farallon	Anton	1		2				3
Rio Cham	Anton			1				1

	Anton total	6		5				11

Rio Atrato	Atrato						9	9
Rio Baudo	Atrato						5	5
Rio San Juan	Atrato						6	6

	Atrato total						20	20

Rio Cascajal	Chagres	4						4
Rio Chagres	Chagres	23						23
Rio Cuango	Chagres	6						6
Rio Pina Pina	Chagres	1						1
Rio Indio	Chagres	6						6

	Chagres total	40						40

Rio Chiriqui	Chiriqui				8			8
Rio Coto	Chiriqui				3			3
Rio San Felix	Chiriqui				1			1

	Chiriqui total				12			12

Rio Cocle del Norte	Cocle del Norte		16					16

Rio Cocle del Sur	Cocle del Sur		6	29				35

Rio Azucar	San Blas					8		8
Rio Mandinga	San Blas					6		6
Rio Playon Chico	San Blas					3		3

	W. San Blas total					17		17

Rio San Pablo	Santa Maria			10				10
Rio Tebario	Santa Maria			1				1
Rio La Villa	Santa Maria			6				6
Rio Oria	Santa Maria			3				3
Rio Santa Maria	Santa Maria			27				27
Rio Tonosi	Santa Maria			2				2

	Santa Maria total			49				49

Rio Bayano	Tuira	54						54
Rio Iglesia	Tuira	4						4
Rio Tuira	Tuira	30						30
Rio Caimito	Tuira	6						6
Rio Capira	Tuira	4						4
Rio Grande	Tuira	2						2
Rio Pacora	Tuira	6						6

	Tuira total	106						106
