# Supplementary material for: Phylogeographic Diversity of the Lower Central American Cichlid Andinoacara coeruleopunctatus (Cichlidae)
Source: Int J Evol Biol. 2012 Sep 12;2012:780169. doi: 10.1155/2012/780169 (PMC3447354; doi:10.1155/2012/780169)
Supplement: Supplementary file 1 — The Supplementary Information file contains tables providing (1) the designation of rivers and drainages into biogeographic provinces (Table S1.1), (2) Genbank accession numbers associated with each sample (pending; Table S1.2), and (3) ATP6/8 haplotype frequencies by river, drainage, and biogeographic region. In addition, the Supplement also provides greater details on (1) choosing the best evolutionary model to use in analyzing the ATP6/8 data set (S1A: Results of jModelTest), (2) additional phylogenetic analyses that were used other than those detailed in the text (S1B: Additional phylogenetic analyses), (3) the resulting phylogeny when an additional 1042bp of ND2 sequence is included with a subset of the ATP6/8 data set (S1C: Additional sequences from ND2 and analyses of the combined datasets), (4) the results for comparison of various migration models using Bayes factors (S1D: Results using Migrate-N), and (5) the results of tests for substitution saturation effects (S1F: Test of saturation ATP6/8). [file 780169.f1.pdf]

Supplementary Information

Table S1.1: Division of rivers and drainages into biogeographic provinces from Smith and Bermingham [4]. Samples from rivers/drainages used in this study are in bold.

Biogeograpic Province	River/drainage
Chiriqui (Pacific)	Terraba, Pirris, San Juan, Palo Blanco, Escarrea, Platanal, **Chiriqui, Coto**, Chriqui Viejo, Chico, Tabasara, **San Felix,** Estero Salado, San Pedro, Cate, IC#26
Santa Maria (Pacific)	Tebario, Tonosi, **Farallon**, **La Villa**, **Santa Maria**, Chorrera, Chame, Parita, **Anton**, Pavo, **Oria**, Playita, Cana, **Cocle del Sur**
Tuira (Pacific)	**Bayano**, Samba, **Tuira**, **Iglesia**, Lara, Cabara, **Caimito**, Juan Diaz, **Pacora**, Capira, Grande, Sajailces
Chagres (Atlantic)	**Mandinga**, **Chargres**, **Azucar**, Cascahal, **Acla**, Cuango, **Cocle del Norte**, Pina Pina, Miguel de la Borda, **Indio**, **Playon Chico**, IC#121

Table S1.2: STRI Freshwater Fish Database identification codes. NOTE: all Genbank Accession numbers pending.

Species		Drainage	STRI id	GenBank ID
A. biseriatus	Colombia	Rio Atrato	stri-113	
			stri-1427	
A. rivulatus	Peru	Rio Canete	stri-15001	
			stri-15002	
		Rio Santa	stri-15010	
			stri-15011	
		Rio Tumbes	stri-15026	
			stri-15027	
		Rio Jequetepeque	stri-15892	
			stri-15893	

A. pulcher	Venezuela	Rio Aroa	VZ-125
		Rio Muyapa	VZ-85
			VZ-86
		Rio Orinoco	VZ-149
	Trinidad	Caroni River	stri-4281
			stri-4288
		Turure River	stri-4255
			stri-4256
A. coeruleopunctatus	Colombia	Rio Atrato	stri-129
			stri-1546
		Rio Baudo	stri-1412
	Costa Rica	Rio Coto	stri-1166
	Panama	Rio Acla	stri-1752
			stri-1753
		Rio Anton	stri-716
		Rio Azucar	stri-3739
		Rio Bayano	stri-2659
			stri-2664
			stri-3625
			stri-3626
			stri-911
		Rio Caimito	stri-4781
			stri-9105
		Rio Chagres	AM-83A
			stri-243
			stri-248
		Rio Chiriqui	AM-267
		Rio Cocle del Norte	AM-23
			AM-58
			stri-1359
		Rio Cocle del Sur	AM-13
			AM-40
			stri-301
		Rio Farallon	stri-3037
		Rio Iglesia	stri-3466
		Rio Indio	stri-2693
		Rio La Villa	stri-1027
		Rio Mandinga	stri-1311
			stri-1312
			stri-1313
		Rio Oria	stri-1048
		Rio Pacora	AM-325
			stri-2721
		Rio Playon Chico	stri-2597

	stri-2635
Rio San Felix	stri-175
Rio Santa Maria	stri-3129
	stri-3168
	stri-3409
	stri-3443
Rio Tuira	AM-216
	AM-217
	stri-3536
	stri-4071
	stri-4072

Table S1.3:

Table of mtDNA ATP6/8 haplotype frequency by river, drainage, and biogeographic region. Excel file available at (NOTE: Due to its size and issue with formatting, the Excel file, and additional data files, will be made available via an FTP site at Wheaton College as soon as possible).

S1A: Results of jModelTest.

The results of model selection using jModelTest [2] suggested eight closely associated models within the 95% CI based on BIC (TrN+G, TrN+I, TrN+I+G, TIM2+G, TIM1+G, HKY+G, TIM2+I, TIM3+G). Of these eight models, only the HKY+G model was not included in the set of twelve models that were members of the 95% CI based on the AIC. The leading model based on the AIC was TIM2+I+G, and the 95% CI set included GTR+I+G as well as the models described above. Both the TrN+G and TIM2+I+G were used in a ML analysis using GARLI [3] with 1000 bootstrap replications. The remaining eight models were assessed using GARLI with 100 bootstrap replications to reduce analysis time in order to compare branching patterns among models.

Near identical trees were obtained regardless of which of the ten models found by jModelTest were used; there were no substantial differences in the relationship or support values among major clades. Therefore we feel there is strong justification for using any

of the following ten models in our phylogenetic analyses: GTR+I+G, TrN+G, TrN+I, TrN+I+G, TIM2+G, TIM1+G, HKY+G, TIM2+I, TIM3+G, and HKY+G).

S1B: Additional phylogenetic analyses

In addition to the ML and Bayesian analyses described in the text, a battery of additional phylogenetic approaches were also performed on the ATP6/8 dataset that varied either in the optimality criteria (minimum evolution, ML, or parsimony) or tree-building algorithm used.

A minimum evolution/maximum likelihood (ME/ML) approach as described in [4] was using the test version 4.0d64 of PAUP*, written by David L. Swofford [5]. Redundant haplotypes were combined into a single OUT, keeping track of geographic origin. We set the optimization criteria as ME using a ML distance measure to estimate the pairwise divergence among haplotypes. The ML model used was the HKY+G with the empirical frequency of nucleotides used to account for differences in nucleotide composition and estimates of the transition/transversion ratio estimated from the data. To further account for variation in rate of divergence among sites, we used an additional model whereby separate rate estimates were made for the three codon positions. An iterative approach was used to estimate all ML model parameters. The starting tree was a Neighbor-Joining (NJ) tree derived from a LogDet paralinear. ML model parameters were estimated from the initial NJ tree, and these estimated parameters used to find a new shortest tree based on ML distance and heuristic methods. The model parameters were re-estimated from this new tree, and the iterative process continued until apparent stability was reached. Support for the resulting shortest trees was inferred by bootstrapping using the final parameter estimates for the ML model. Bootstrapping was performed using 1000 randomizations and the fast-addition option found in PAUP*.

Additional maximum likelihood analyses was performed using PUZZLE v4.0 [6]. Branch lengths, their standard errors, and the quartet puzzling support for each branch was determined with site variation accounted for using the gamma distribution parameter (estimated from the data) and 1000 quartet puzzling steps.

A parsimony analysis was also run using three different weighting schemes using the program PAUP*: equal weighting, weighting transitions 1/6 of transversions and by

codon position (3:9:1), and Farris' succesive approximations [7]. Branch support was assessed by bootstrapping (1000 randomizations) using the fast step option.

Finally, minimum evolution analyses were performed using MEGA [8] using a variety of distance measures, topology estimation, and clade confidence estimated by bootstrapping and interior-branch tests.

Nearly equivalent trees to Figure 2 were found for all of the above analyses when taking bootstrap, interior-branch test, or other support values into consideration. For brevity's sake, details from any one analysis will not be presented here, but can be obtained from SSMc upon request. We therefore consider Figure 2 a robust, conservative representative of the ATP6/8 phylogeny.

S1C: Additional sequences from ND2 and analyses of the combined datasets.

Since the relationship among many of the principal clades could not be resolved with the ATP6/8 data, an additional 1047 bp of ND2 sequence was collected from a representative subsample of 2 individuals from each of the primary clades and analyzed using the methods described in the text including partitioning the data into separate gene regions and codon positions in an effort to resolve internal branching order. The primer pair MET/ASN [9] was used for amplification following routine conditions, with primers ND2.1aeq (5'-GCYYTAGCAMTAAAAATTGG-3') and ND2.3aeq (5'-TATTTTRTTAT ACMACCTC-3') were used for internal sequencing. In total, we sequenced the complete ND2 gene for 23 ingroup and outgroup individuals representative of the mtDNA clades defined by the ATP6/8 data. The final dataset consisted of a total of 1899 base pairs, of which 410 were parsimony informative.

The program jModelTest [6] was used on the combined ATP6/8 and ND2 dataset with partitioning. A partition analysis of the combined ATP6/8-ND2 dataset was performed using GARLI [7] using the TRN+I+G and GTR+I+G models for the ATP6/8 and ND2 data partitions respectively and 1000 bootstrap replications. A partitioned Baysian analysis using the above two models was performed using MrBayes as described in the text for the ATP6/8 dataset.

Figure S1C.1: Phylogenetic relationship among mtDNA haplotypes. On the left (A) is the ATP6/8 ML tree as described in text, on right (B) is the combined ATP6/8 and ND2 tree. OTUs are identified by drainage or biogeographic region according to Smith and Bermingham [1]. Bootstrap support values for the major clades are above the branches, posterior probabilities from MrBayes below. Branch colors reflect biogeographic regions or drainage areas as found in Figure 1. The inset map (C) is a stylized representation of the range of the major clades along drainage and biogeographic boundaries.

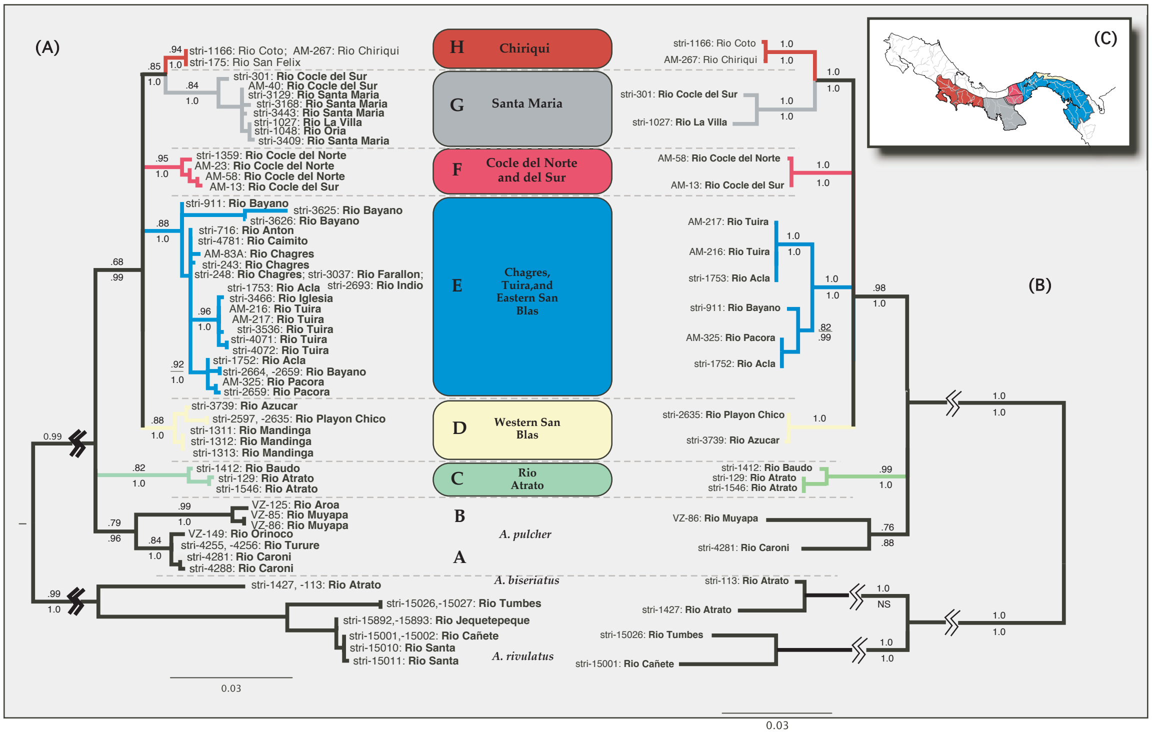

S1D: Results using Migrate-N

To test various hypotheses of migration between biogeographic regions, we used the program Migrate-n [10] using the marginal probabilities (Bayes factors) to test specific hypotheses. Three models were routinely tested between two regions: a full

migration model, migration from region 1 to region 2 only, and from region 2 to region 1 only. Initial estimates of priors were made by maximum likelihood. Repeated runs of varying lengths (from 10^6 to 10^7) and increasing the number of long chains and heating were used. In all models, k (transition/transversion ratio) was set at 13.8 as estimated from PAUP* [5]. Priors for theta and M were set using a uniform random prior with a range from 0 to twice the estimated value from the ML analysis. Different values and distributions for the priors of theta and M were also tried. Usual settings for MCMC analysis was 10^7 generations with a burn-in of 100,000, running four heated chains with static value set at 1, 1.5, 30, and 100000.

In all cases, for all analyses tried, the program failed to find optimal estimates for either theta or M (though theta was usually reliably estimated and M distribution plots were similar among runs) and did not appear to approach stationarity despite adjusting priors, number of generations, or number of long or heated chains. The following figure is representative of the results from many of the runs. Therefore we consider these results highly suspect, and discuss them here for completeness sake only.

Figure: Example Posterior distribution of parameters from the full migration model, CdN/CdS. Notice the general lack of a specific peak for estimates $M_{1->2}$.

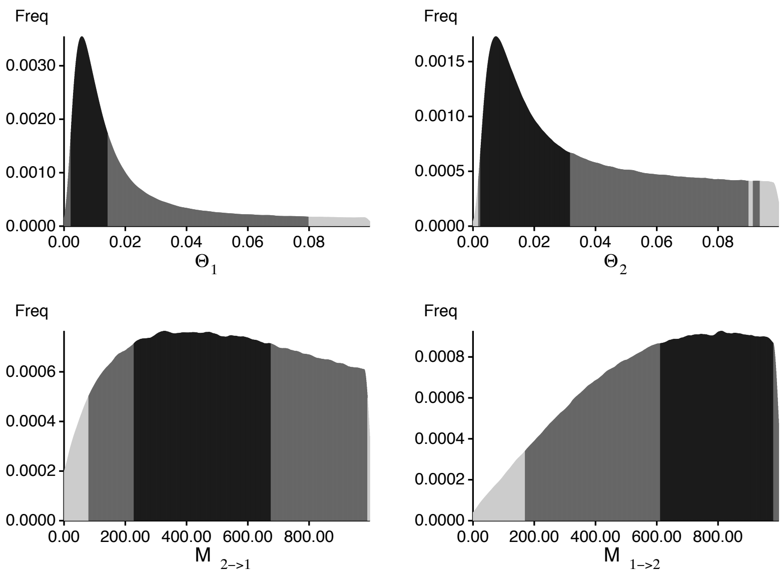

One possible use of these analyses, rather than providing specific estimates of Theta or M, is to test the three different migration models using Bayes factors in order to determine which model has the greatest Bayesian support. The results for the migration models testing for CdN-CdS and TUI-CHA are given below as estimates of the mean of the parameters of theta and M and the likelihood values for each model used in the calculations for marginal probabilities of each model (Bayes factor).

CdN-CdS Migration Model Test Results

Table of posterior values for the three models

	Full Model	CdS -> CdN	CdN -> CdS
$\cup_1$	0.02317	0.01931	0.05274
$\cup_2$	0.03843	0.05096	0.02240
$M_{2\to1}$	513.9	–––	707.0
$M_{1\to2}$	606.9	608.2	–––

Table of likelihood values for each model used in the Bayes factor analyses

Model	Bezier	IBL	Model prob
**CdN-CdS**			
**full**	-1344.65	0.12653	0.09785
**S -> N**	-1342.58	1	0.77333
**N -> S**	-1344.37	0.16656	0.12880

The above table shows that the highest support was for a model of one-way migration from CdS to CdN, contrary to the ML results and our intuitive interpretation of one way migration from CdN to CdS.

Summary of Migrate results: As discussed in the text, our interpretation of these results is that our dataset is simply insufficient for this type of analysis, and that the results from the Bayes factor approach should be viewed with caution. Nonetheless determining the migration patterns in this region is important to understanding the history or migration in this important region of Panama. If migration has been from CdS to CdN only as implied by the Bayes factor approach, then the implication is that CdS is the source of CdN *A. coeruleopunctatus*. This would suggest the evolution of an early CdS endemic clade that subsequently colonized CdN via stream capture. CdS was subsequently colonized by neighboring Santa Maria (SM) clades via anastomosis resulting in two highly divergent clades currently segregating in CdS (we assume this scenario as the most parsimonious given the relative frequency of haplotypes in the CdS and CdN, though agree that an alternative explanation that CdN was colonized by only one of two cosegregating haplotypes in CdS is also plausible). In contrast our intuitive interpretation is that CdN clades evolved in isolation in Cocle del Norte, most likely derived from a common ancestor with Charges/Tuira clades, and subsequently colonized Cocle del Sur and are currently co-segregating with Santa Maira derived lineages.

CHA-TUI Migration Model Test

Similar evidence for cross cordillera exchange was also detected in the Tuira (TUI) and Chargres (CHA) regions. To test for alternative models of migration between these biogeorgaphic provinces, we used Bayes factors to test for which of three competing migration models in this region (full migration model, CHA to TUI only, and TUI to CHA only) had the highest probability. All Bayes analyses were performed as described above. Though these results are not discussed in the text, they are presented here from completeness sake.

Table of posterior values for the three migration models

	Full Model	CHA -> TUI	TUI -> CHA
U_1	0.01227	0.01497	0.02836
U_2	0.03164	0.05057	0.01181
$M_{2\text{->}1}$	534.3	---	756.2
$M_{1\text{->}2}$	678.7	780.4	---

Table of likelihood values for each model used in the Bayes factor analyses

Model	Bezier	IBL	Model prob
**Full**	-1580.91	0.045452	0.02681
**CHA -> TUI**	-1578.26	0.649382	0.38315
**TUI -> CHA**	-1577.82	1	0.59002

The above table shows that the highest support was for a model of one-way migration from Tuira to Chargres.

S1E: Results using BEAST

DETAILS PENDING COMPILATION AND SUMMARY

S1F: Test of saturation ATP6/8

Substitution saturation in the ATP6/8 dataset was tested following Xia [11] using the program DAMBE 5.3 [12] with the number of replicates set to 100 and all sites included. Table S1F.1 presents the results from the DAMBE output. In all instances Iss was significantly lower than Iss.C (p=0.000, 841df) assuming both a symmetrical and nonsymmetrical phylogeny [13] demonstrating no substantial saturation effects.

Table S1F.1: Results of test for saturation effects following [11].

NumOTU	Iss	Iss.c Sym	T	DF	P	Iss.c Assym	T	DF	P
4	0.065	0.815	87.259	851	0.0000	0.783	83.588	851	0.0000
8	0.069	0.780	76.424	851	0.0000	0.672	64.868	851	0.0000
16	0.070	0.762	73.608	851	0.0000	0.558	51.967	851	0.0000
32	0.070	0.736	71.765	851	0.0000	0.422	37.848	851	0.0000

NOTE: Two-tailed tests were used

Similar evidence for a lack of saturation effects can be seen graphically in Figure S1A.1, which shows a plot of Estimated ML Divergence (using the model described in text) against uncorrected levels of divergence for both the ATP6/8 and ND2 (see below) datasets [14]. Notice the lack evidence of a nonlinear relationship for all ingroup comparisons, and little evidence from comparison with outgroup.

Figure S1F.1: Plot of pairwise Estimated ML Divergence versus uncorrected divergence for ATP6/8 and ND2 datasets.

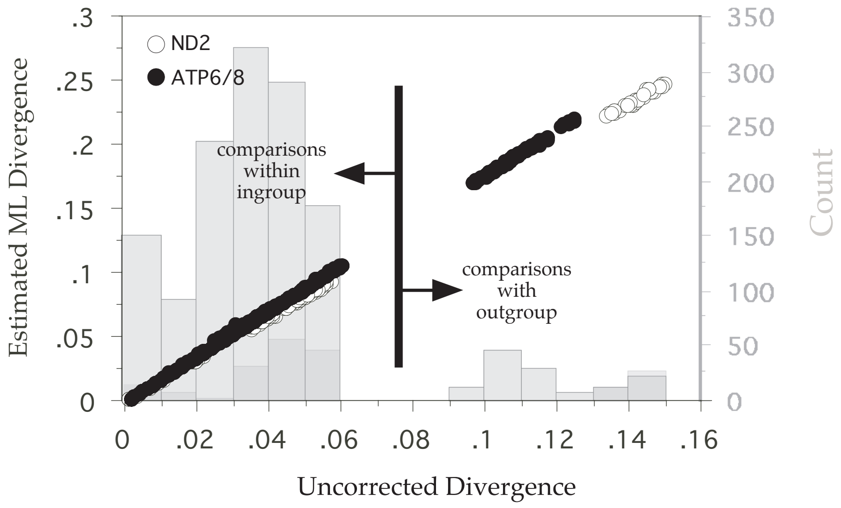

References

1. S. Smith and E. Bermingham, "The biogeography of lower Mesoamerican freshwater fishes," Journal of Biogeography, vol. 32, pp. 1835-1854, 2005.

2. D. Posada, "jModelTest: Phylogenetic Model Averaging," Molecular Biology and Evolution, vol. 25, pp. 1253-1256, 2008.

3. D.J. Zwickl, "Genetic algorithm approaches for the phylogenetic analysis of large biological sequence datasets under the maximum likelihood criterion," Ph.D. dissertation, The University of Texas at Austin, 2006.

4. S. McCafferty, E. Bermingham, B. Quenouille, S. Planes, G. Hoelzer, and K. Asoh, "Historical biogeography and molecular systematics of the Indo-Pacific genus *Dascyllus* (Teleostei: Pomacentridae)," Molecular Ecology, vol. 11, pp. 1377-92, 2002.

5. D.L. Swofford, "PAUP* Phylogenetic analysis using parsimony (*and other methods) Version 4.0d64," Sinauer Associates, Sunderland, MA, 1998.

6. K. Strimmer, and A. von Haeseler, "Quartet puzzling: a quartet maximum likelihood method for reconstructing tree topologies," Molecular Biology and Evolution, vol. 13, pp. 964-969, 1996.

7. J.S. Farris, "A successive approximations approach to character weighting," Systematic Zoology, vol. 18, pp. 374-385, 1969.

8. K. Tamura, J. Dudley, M. Nei, and S. Kumar, "MEGA4: Molecular Evolutionary Genetics Analysis (MEGA) software version 4.0," Molecular Biology and Evolution, vol. 24, pp 1596-1599, 2007.

9. T.D. Kocher, J.A. Conroy, K.R. McKaye, J.R. Stauffer, and S.F. Lockwood, "Evolution of NADH dehydrogenase subunit 2 in East African cichlid fish," Molecular Phylogenetics and Evolution, vol. 4, pp. 420-432, 1995.

10. P. Beerli and J. Palczewski, "Unified Framework to Evaluate Panmixia and Migration Direction Among Multiple Sampling Locations," Genetics, vol. 185 pp. 313—326, 2010.

11. X. Xia, Z. Xie, M. Salemi, I. Chen, and Y. Wang, "An index of substitution saturation and its implication," Molecular Phylogenetics and Evolution, vol. 26, pp. 1-7, 2003.

12. X. Xia, and Z. Xie, "DAMBE: Data analysis in molecular biology and evolution," Journal of Heredity, vol. 92, pp. 371-373, 2001.

13. X. Xia, and P. Lemey, "Assessing substitution saturation with DAMBE", *The Phylogenetic Handbook: A Practical Approach to DNA and Protein Phylogeny*

2nd Ed., P. Lemey, M. Salemi, and A Vandamme Eds, Cambridge University Press, Cambridge, England, 2009.

14. H. Philippe, "MUST, a computer package of management utilities for sequences and trees," Nucleic Acid Research, vol. 21, pp. 5264-5272, 1993.
